# HIV- and HCV-specific markers and echocardiographic pulmonary artery systolic pressure among United States veterans

**DOI:** 10.1038/s41598-020-75290-4

**Published:** 2020-10-30

**Authors:** Courtney E. Zola, Meredith S. Duncan, Kaku So-Armah, Kristina A. Crothers, Adeel A. Butt, Cynthia L. Gibert, Joon Woo W. Kim, Joseph K. Lim, Vincent Lo Re, Hilary A. Tindle, Matthew S. Freiberg, Evan L. Brittain

**Affiliations:** 1 Division of Infectious Disease, Department of Medicine, Vanderbilt University Medical Center, Nashville, TN USA; 2 Division of Cardiovascular Medicine, Vanderbilt University Medical Center, 2525 West End Avenue, Suite 300A, Nashville, TN 37203 USA; 3 School of Medicine, Section of General Internal Medicine, Boston University, Boston, MA USA; 4 Department of Medicine, University of Washington School of Medicine, Seattle, WA USA; 5 VA Pittsburgh Healthcare System, Pittsburgh, PA USA; 6 Weill Cornell Medical College, New York, NY USA; 7 Weill Cornell Medical College, Doha, Qatar; 8 Department of Medicine, George Washington University, Washington, DC USA; 9 Department of Medicine, Icahn School of Medicine At Mt. Sinai, James J. Peters VA Medical Center, New York City, NY USA; 10 Department of Medicine, Yale University School of Medicine, New Haven, CT USA; 11 Division of Infectious Disease, Department of Medicine and Center for Clinical Epidemiology and Biostatistics, Perelman School of Medicine, University of Pennsylvania, Philadelphia, PA USA; 12 Geriatric Research Education and Clinical Centers (GRECC), Veterans Affairs Tennessee Valley Healthcare System, Nashville, TN USA; 13 Department of Medicine, Vanderbilt University Medical Center, Nashville, TN USA

**Keywords:** Cardiology, Risk factors

## Abstract

Hepatitis C virus (HCV) may increase pulmonary hypertension (PH) risk among people living with HIV (PLWH). Prior studies on this topic have been relatively small and examined selected populations. We determine whether HIV/HCV coinfection is associated with higher pulmonary artery systolic pressure (PASP) and prevalent echocardiographic PH. We performed a cross-sectional analysis of 6032 (16% HIV/HCV coinfected) Veterans Aging Cohort Study participants enrolled 4/1/2003–9/30/2012 with echocardiographic PASP measures. We performed multiple linear and logistic regression analyses to determine whether HIV/HCV mono- or co-infection were associated with PASP and PH compared to uninfected individuals. Individuals with HIV/HCV coinfection displayed a higher PASP than uninfected individuals ($\widehat{\beta }$=1.10, 95% CI 0.01, 2.20) but there was no association between HIV/HCV coinfection and prevalent PH. Subset analyses examined HIV and HCV disease severity markers separately and jointly. Among PLWH, HCV coinfection ($\widehat{\beta }$=1.47, 95% CI 0.26, 2.67) and CD4 + cell count ($\widehat{\beta }$= − 0.68, 95% CI − 1.10, − 0.27), but not HIV viral load nor ART regimen, were associated with PASP. Among people with HCV, neither HIV coinfection nor HCV biomarkers were associated with PASP. Among US veterans referred for echocardiography, HIV/HCV coinfection was not associated with a clinically significant elevation in pulmonary pressure. Lower absolute CD4 + T-cell count was inversely associated with PASP which warrants further investigation in prospective studies.

## Introduction

People living with HIV (PLWH) are at increased risk for pulmonary hypertension (PH), compared to uninfected individuals^1,2^. Smaller cohorts examining HIV-associated PH have yet to conclusively demonstrate whether HIV-specific factors contribute to pulmonary pressure or if comorbidities (e.g., heart failure and chronic obstructive pulmonary disease) drive PH risk among individuals with HIV^3^.

Small studies in selected populations suggest that the prevalence of PH may be increased among PLWH coinfected with chronic hepatitis C virus (HCV) coinfection^4–7^; these observations have yet to be examined in large cohorts. Moreover, the associations between HCV viral replication and pulmonary artery systolic pressure (PASP) have not been well-described.

The primary goal of this study is to determine whether HIV/HCV coinfection is associated with echocardiographic PH among US veterans and to identify viral factors associated with higher PASP. We hypothesized that HIV/HCV coinfection would be associated with echocardiographic PH and that markers of uncontrolled HIV/HCV disease activity would be associated with higher echocardiographic estimates of PASP. To test these hypotheses, we used data from the Veterans Aging Cohort Study (VACS), an electronic health record-based cohort that contains demographic, clinical, and echocardiographic data on veterans with and without HIV infection^8^. This study builds on our prior work in VACS examining the effects of viral load, CD4 + count, and HCV on PASP^9,10^.

## Results

### Cohort Characteristics

Among 6032 VACS participants who met all inclusion criteria, 2795 were uninfected, 1402 had HIV mono-infection, 846 subjects had HCV mono-infection and 989 were HIV/HCV coinfected. The four groups had comparable age and sex distributions; participants were 57 years of age, on average, and 97% of the sample was male. The prevalence of African American race was higher in the HCV only and HIV/HCV coinfection groups (64% and 61%, respectively) as compared to the uninfected and HIV mono-infected groups (46% and 44%, respectively). The uninfected group had a significantly higher average BMI (30.2 ± 6.8) when compared to the three infected groups (average BMI range 25.5–27.8). Regarding chronic comorbid conditions, HIV mono-infection and HIV/HCV coinfection were associated with lower rates of diabetes and hypertension. History of coronary heart disease was most common among the uninfected (52%). Stroke and congestive heart failure rates were similar across groups (Table 1).

**Table 1:** Group characteristics stratified by infection status.

	Uninfected	HIV Only	HCV Only	HIV/HCV
	(n = 2795)	(n = 1402)	(n = 846)	(n = 989)
**Age, years**	57.8 ± 9.5	57.4 ± 10.7	56.2 ± 6.1	56.2 ± 7.4
**Race**				
Caucasian	1110 (39.7)	617 (44.0)	221 (26.1)	283 (28.6)
African American	1294 (46.3)	615 (43.9)	545 (64.4)	598 (60.5)
Hispanic	328 (11.7)	113 (8.1)	73 (8.6)	92 (9.3)
Other	63 (2.3)	57 (4.1)	7 (0.8)	16 (1.6)
**Male Sex**	2694 (96.4)	1362 (97.2)	834 (98.6)	966 (97.7)
**BMI, kg/m**^2^^†^	30.2 ± 6.8	26.1 ± 5.7	27.8 ± 6.1	25.5 ± 5.3
**PASP, mmHg**	35.6 ± 14.4	35.1 ± 14.5	36.6 ± 14.6	37.0 ± 16.2
**Heart failure status**				
None	1900 (68.0)	1017 (72.5)	597 (70.6)	707 (71.5)
HF Preserved EF	144 (5.2)	53 (3.8)	45 (5.3)	60 (6.1)
HF, EF between 40 & 50	52 (1.9)	34 (2.4)	12 (1.4)	27 (2.7)
HF Reduced EF	174 (6.2)	116 (8.3)	58 (6.9)	75 (7.6)
HF, No EF	525 (18.8)	182 (13.0)	134 (15.8)	120 (12.1)
**CHD History**	1450 (51.9)	577 (41.2)	387 (45.7)	318 (32.2)
**Hypertension**^**†**^				
Absent	99 (3.5)	144 (10.3)	30 (3.6)	75 (7.6)
Controlled HTN	1842 (65.9)	924 (65.9)	506 (59.9)	616 (62.3)
Uncontrolled HTN	854 (30.6)	334 (23.8)	309 (36.6)	298 (30.1)
COPD*	949 (34.0)	452 (32.2)	312 (36.9)	348 (35.2)
Portal Hypertension*	17 (0.7)	3 (0.2)	35 (4.1)	28 (2.8)
Stroke History*	213 (7.6)	91 (6.5)	69 (8.2)	60 (6.1)
**Diabetes**	1284 (45.9)	469 (33.5)	390 (46.1)	385 (38.9)
**Smoking status**^**†**^				
Never	907 (34.7)	440 (34.8)	143 (17.8)	200 (21.7)
Former	680 (26.0)	281 (22.3)	182 (22.6)	226 (24.5)
Current	1025 (39.2)	542 (42.9)	479 (59.6)	497 (53.9)
**Dyslipidemia**^†^	1638 (59.3)	949 (69.2)	472 (57.9)	635 (66.0)
**Alcohol abuse**	913 (32.7)	396 (28.3)	545 (64.4)	535 (54.1)
**Cocaine abuse**	441 (15.8)	272 (19.4)	384 (45.4)	443 (44.8)
**eGFR,** **mL/min/1.73 m**^**2**^^†^	79.7 ± 31.3	79.9 ± 34.9	84.9 ± 46.9	82.3 ± 39.1
**FIB-4**^†^	1.5 ± 1.5	1.8 ± 2.7	2.7 ± 3.5	3.2 ± 3.7
**Hemoglobin,** **mg/dL**^†^	13.6 ± 1.9	13.1 ± 2.0	13.3 ± 2.1	12.9 ± 2.1
**HIV RNA, copies/mL**^**†**^		36,842.2 ± 237,295.7		24,558.8 ± 85,996.9
Median		75.0		75.0
**CD4 + T-cell count, cells/mm**^**3†**^		451.9 ± 305.2		417.2 ± 275.8
Median		394.0		364.0
**Nadir CD4 + T-cell count, cells/mm**^3^*,^**†**^		259.5 ± 210.8		241.9 ± 184.1
Median		215.0		209.0
**ART regimen **				
NRTI + PI		294 (21.0)		207 (20.9)
NRTI + NNRTI		222 (15.8)		106 (10.7)
Other regimen		653 (46.6)		504 (51.0)
No ART		233 (16.6)		172 (17.4)
HCV RNA, copies/mL*,^**†**^			1,769,144.4 ± 3,806,313.0	2,550,873.6 ± 5,854,693.8
Median			500,000	604,000
**HCV treatment **				
Interferon			24 (2.8)	21 (2.1) 21 (2.1)
Ribavirin			21 (2.5)	21 (2.1)
Telaprevir*			1 (0.1)	0 (0.0)

Data presented as mean ± standard deviation or n(%). unless otherwise specified

*SD* standard deviation; *BMI* body mass index, *PASP* pulmonary artery systolic pressure, *HF* heart failure, *EF* ejection fraction, *CHD* coronary heart disease, *HTN* hypertension, *COPD* chronic obstructive pulmonary disease, *eGFR* estimated glomerular filtration rate, *FIB-4* fibrosis 4 score, *RNA* ribonucleic acid, *ART* anti-retroviral therapy, *NRTI* nucleoside reverse transcriptase inhibitor, *PI* protease inhibitor, *NNRTI* non-nucleoside reverse transcriptase inhibitor, *HCV* hepatitis C virus.

HF preserved EF is defined as prevalence of HF with EF > 50; HF reduced EF is defined as prevalence of HF with EF < 40.

*All characteristics were significantly different across HIV/HCV groups, via Wilcoxon tests or χ^2^ test except COPD (*p* = 0.1330), portal hypertnsion (*p* < 0.0001), history of stroke (*p* = 0.1821), CD4 Nadir (*p* = 0.3113), HCV viral load (*p* = 0.2710), and telaprevir (*p* = 0.1054).

^†^All variables had complete data except BMI (available on 2795 uninfected, 1402 HIV mono-infected, 844 HCV mono-infected, 987 co-infected), hypertension (available on 2795 uninfected, 1402 HIV mono-infected, 845 HCV mono-infected, 989 co-infected), smoking status (available on 2612 uninfected, 1263 HIV mono-infected, 804 HCV mono-infected, 923 co-infected), dyslipidemia (available on 2764 uninfected, 1371 HIV mono-infected, 815 HCV mono-infected, 962 co-infected), eGFR (available on 2790 uninfected, 1401 HIV mono-infected, 846 HCV mono-infected, 989 co-infected), FIB-4 (available on 2720 uninfected, 1228 HIV mono-infected, 836 HCV mono-infected, 872 co-infected), hemoglobin (available on 2784 uninfected, 1402 HIV mono-infected, 846 HCV mono-infected, 987 co-infected), HIV RNA (available on 1217 HIV mono-infected, 873 co-infected), CD4 cell count (available on 1233 HIV mono-infected, 874 co-infected), CD4 nadir (available on 1233 HIV mono-infected, 874 co-infected), and HCV RNA (available on 655 HCV mono-infected, 795 co-infected).

The mean PASP for the entire cohort was 35.8 ± 14.8 mmHg and was highest in the HIV/HCV coinfection group (37.0 ± 16.2 mmHg). In total, 40.3% of individuals had a PASP greater than 35 mmHg, 26.2% had PASP greater than 40 mmHg, and 4.9% had PASP greater than 60 mmHg.

Among PLWH, those with mono-infection had a median CD4 of 394 cells/mm^3^ versus those with HIV/HCV coinfection whose mean CD4 cell count was lower at 364 cells/mm^3^, but both groups had a median baseline HIV viral load of 75 copies/mL. In addition, men had lower median CD4 cell counts (380 cells/mm^3^) than women (497 cells/mm^3^); median HIV viral load was the same in both sexes (75 copies/mL). In the HCV + groups, individuals with mono-infection had a median HCV viral load of 500,000 copies/mL while those with HIV/HCV coinfection had a much higher median HCV viral load of 604,000 copies/mL.

### Regression analysis

In regression analyses, HIV/HCV coinfection was associated with elevated PASP compared to neither infection (*p* = 0.048), while neither HIV mono-infection (*p* = 0.99) nor HCV mono-infection (*p* = 0.43) were associated with increased PASP compared to neither infection (Table 2). Compared to white race, African American race was associated with higher PASP ($\widehat{\beta }$= 2.02 [1.20, 2.85], *p* < 0.001) while Hispanic ethnicity was associated with lower PASP ($\widehat{\beta }$= − 3.31 [− 4.56, − 2.06], *p* < 0.001). Male sex was associated with higher PASP ($\widehat{\beta }$= 7.40 [5.31, 9.50], *p* < 0.001), although the female sample was small. History of heart failure, heart disease, COPD, and diabetes mellitus were associated with increased PASP. Decreased kidney function (decreased eGFR), liver cirrhosis (increased FIB-4), and anemia (decreased hemoglobin) were also associated with increased PASP (Table 2).

**Table 2:** Linear regression of PASP, entire cohort.

Variables	β-estimate (mmHg) [95% CI]*	*p* value
**HIV/HCV status**		
Uninfected	Ref	–
HIV mono-infected	0.002 [− 0.93, 0.93]	0.9963
HCV mono-infected	0.45 [− 0.66, 1.57]	0.4259
Co-infected	1.10 [0.01, 2.20]	0.0484
**Age, 10 years**	0.21 [− 0.22, 0.63]	0.3453
**Race**		
White	Ref	–
African American	2.02 [ 1.20, 2.85]	< .0001
Hispanic	− 3.31 [− 4.56, − 2.06]	< .0001
Other	0.16 [− 2.17, 2.49]	0.8953
**Male Sex**	7.40 [5.31, 9.50]	< .0001
**BMI**	0.04 [− 0.02, 0.10]	0.2436
**Heart failure status**		
HF Preserved EF	7.83 [6.18, 9.48]	< .0001
HF with EF between 40 & 50	6.46 [4.00, 8.92]	< .0001
HF Reduced EF	5.78 [4.37, 7.18]	< .0001
No EF	7.94 [6.90, 8.98]	< .0001
No HF	Ref	–
**History of CHD**	1.02 [0.24, 1.80]	0.0102
**Hypertension**		
No Hypertension	Ref	–
Controlled HTN	0.31 [− 1.27, 1.88]	0.7003
Uncontrolled HTN	1.65 [− 0.02, 3.31]	0.0523
**COPD**	1.96 [1.19, 2.72]	< .0001
**Stroke History**	− 1.19 [− 2.55, 0.16]	0.0844
**Diabetes Mellitus**	0.89 [0.13, 1.66]	0.0214
**Dyslipidemia**	− 0.44 [− 1.18, 0.30]	0.2480
**Smoking status**		
Current	0.46 [− 0.50, 1.43]	0.3438
Former	0.91 [− 0.19, 2.02]	0.1049
Never	Ref	–
**Alcohol Use**	0.21 [− 0.67, 1.09]	0.6346
**Cocaine Use**	− 1.53 [− 2.55, − 0.50]	0.0034
**eGFR**	− 0.03 [− 0.04, − 0.01]	< .0001
**FIB-4**	0.31 [0.17, 0.45]	< .0001
**Hemoglobin**	− 0.75 [− 0.94, − 0.56]	< .0001

*PASP* pulmonary arter systolic pressure, *HCV* hepatitis C virus, *CI* confidence interval, *BMI* body mass index, *HF* heart failure, *EF* ejection fraction, *CHD* coronary heart disease, *HTN* hypertension, *COPD* chronic obstructive pulmonary disease, *eGFR* estimated glomerular filtration rate; *FIB-4* fibrosis 4 score.

*All estimates are from a multiple linear regression adjusted for all listed variables. Estimates per one unit increase unless otherwise specified.

In models restricted to PLWH, we observed that HCV coinfection and lower CD4 + cell count were associated with increased PASP (${\widehat{\beta }}_{HCV}$= 1.47 [0.26, 2.67], *p* = 0.0170; ${\widehat{\beta }}_{200 CD4+ Cells}$ =  − 0.68 [− 1.10, − 0.27], *p* = 0.0011). However, we did not observe significant associations of HIV viral load or ART regimen with increasing PASP levels (Table 3). In models restricted to individuals with HCV infection, we did not observe significant associations of HIV coinfection, HCV viral load, or interferon exposure with increasing PASP levels. However, analyses of interferon exposure may have suffered from a lack of statistical power due to the reduced sample size and low uptake.

**Table 3:** Association of HIV/HCV viral markers and medication regimens with pASP (mmHg).

	β-estimate (mmHg) [95% CI]*	*p* value
**Variables among All HIV + **		
HCV positive status	1.47 [0.26, 2.67]	0.0170
HIV viral load (10,000 copies/mL)	− 0.02 [− 0.05, 0.02]	0.3481
CD4 + T-cell count (200 cells/mm^3^)	− 0.68 [− 1.10, − 0.27]	0.0011
**ART regimen**		
NRTI + PI vs. No ART	0.65 [− 1.28, 2.59]	0.5088
NRTI + NNRTI vs. No ART	0.53 [− 1.59, 2.65]	0.6258
Other ART vs. No ART	− 0.90 [− 2.59, 0.79]	0.2981
**Variables among All HCV cases**		
HIV positive status	0.53 [− 0.99, 2.05]	0.4922
HCV viral load (10,000 copies/mL)	0.001 [− 0.0003, 0.003]	0.1084
Interferon exposure	0.70 [− 3.74, 5.13]	0.7578

*HCV* hepatitis C virus, *PASP* pulmonary artery systolic pressure, *CI* confidence interval, *ART* anti-retroviral therapy, *NRTI* nucleoside reverse transcriptase inhibitor, *PI* protease inhibitor, *NNRTI* non-nucleoside reverse transcriptase inhibitor.

*Adjusted for: age, sex, race/ethnicity, and heart failure.

In logistic regression analyses, we did not observe significant associations between prevalent echocardiographic PH and HIV mono-infection, HCV mono-infection, or HIV/HCV coinfection versus neither infection at clinically relevant estimated PASP cutoffs—35 mmHg, 40 mmHg, or 60 mmHg (Table 4). While neither HIV nor HCV were associated with echocardiographic PH, female sex was associated with lower odds of echocardiographic PH than male sex for the 35 mmHg (Odds Ratio (OR) [95% CI]: 0.44 [0.30–0.65]) and 40 mmHg (OR [95% CI]: 0.36 [0.22–0.60]) cutoffs and African American race was consistently associated with higher odds of echocardiographic PH than white race (OR_35mmHg_ [95% CI]: 1.28 [1.13–1.45]; OR_40mmHg_ [95% CI]: 1.38 [1.20–1.59]; OR_60mmHg_ [95% CI]: 1.44 [1.07–1.94]).

**Table 4:** Association of common clinical PASP cutoffs and HIV, HCV infection status.

	Odds Ratio [95% CI]* vs. uninfected	*p* value
**Association with PASP > 35 mmHg**		
HIV mono-infection	0.95 [0.86,1.06]	0.3669
HCV mono-infection	1.04 [0.92, 1.18]	0.5022
HIV/HCV Coinfection	1.08 [0.96, 1.21]	0.2262
**Association with PASP > 40 mmHg**		
HIV mono-infection	1.00 [0.89, 1.13]	0.9905
HCV mono-infection	0.99 [0.86, 1.13]	0.8521
HIV/HCV Coinfection	1.10 [0.97, 1.25]	0.1455
**Association with PASP > 60 mmHg**		
HIV mono-infection	1.01 [0.80, 1.27]	0.9405
HCV mono-infection	1.00 [0.77, 1.30]	0.9981
HIV/HCV Coinfection	1.22 [0.97, 1.55]	0.0954

*PASP* pulmonary artery systolic pressure, *HCV* hepatitis C virus, *CI* confidence interval.

*Adjusted for: age, sex, race, hypertension, diabetes, dyslipidemia, smoking status, estimated glomerular filtration rate, body mass index, hemoglobin concentration, FIB-4 (fibrosis-4) score, alcohol abuse, cocaine abuses, chronic obstructive pulmonary disease status, heart failure, stroke history, and heart disease history.

### Sensitivity and exploratory analyses

Results from sensitivity analyses including those of unknown HCV status in the HCV negative groups yielded nearly identical results (Tables S1–S3). In exploratory analyses limited to individuals with HIV/HCV coinfection, prevalent portal hypertension was associated with higher PASP ($\widehat{\beta }$= 7.21 [1.11, 13.31], *p* = 0.0205) but not with prevalent echocardiographic PH using any threshold.

## Discussion

The rationale for this study was to examine, in a large and unselected population, the relationship between HIV/HCV co-infection and echocardiographic PASP. In this unselected cohort of veterans referred for clinically indicated echocardiograms, HIV/HCV coinfection was associated with higher values of absolute PASP but coinfection was not associated with prevalent PH using common echocardiographic thresholds. In PLWH, we observed a modest inverse association between absolute CD4 + T-cell count and higher PASP estimates, while absolute HIV viral load was not associated with higher PASP. Other notable findings include lack of an association between ART regimen or interferon exposure with PASP and a positive association between African American race and prevalent PH.

Our study has a few key differences from previous studies examining PH among individuals with HIV and/or HCV. Our cohort underwent clinically-indicated echocardiograms as opposed to screening studies. As such, our cohort is older and has a higher prevalence of cardiopulmonary comorbidities (e.g. HF and COPD), which may explain the higher prevalence of PASP > 35 mmHg (40% vs. 5–23%)^7,11,12^. The high prevalence of HF and COPD in particular likely contributes to the relative high prevalence of echocardiographic PH in our cohort; however, our statistical models account for these comorbidities, among others. Another retrospective analysis using clinically-indicated studies reported a prevalence of PH or RV dysfunction of 26%^5^. Ours is the largest (by an order of magnitude) echocardiographic cohort to examine specific HIV and HCV viral markers and PASP as continuous variables. Previous studies have been small (allowing for limited adjustment for confounders) or evaluated HIV-associated PH using HIV classifications such as HIV stage or CD4 + T-cell count < 200 and PH cutoffs of PASP > 35 mmHg^2,5^.

Our study is the largest to describe the prevalence of echocardiographic PH among individuals with HIV/HCV coinfection and examine the association of HIV/HCV disease markers with PH and the only to include HIV and HCV mono-infected cohorts as comparators. The prevalence of PASP > 40 mmHg among 989 HIV/HCV co-infected veterans in our cohort was 29%, which did not differ significantly from the mono-infected or uninfected groups. We found a modest association between HIV/HCV coinfection and PASP ($\widehat{\beta }$ = 1.10 [0.01, 2.20], *p* = 0.048) but the clinical relevance of this result is unclear as coinfection was not associated with prevalent echocardiographic PH in logistic regression analyses. Previous publications have reported the prevalence of PH among individuals with HIV/HCV coinfection. Reiberger et al. reported a PH prevalence of 10% (mean PA pressure ≥ 20 mmHg) among 80 selected individuals undergoing invasive evaluation in the context of a clinical trial^6^. This study did not include any control groups against which to compare the prevalence of PH and did not examine the effects of HIV and HCV viral activity on pulmonary pressure. In a retrospective analysis, Sangal et al. reported a PH prevalence (defined as PASP > 40 mmHg) of 19% among 43 individuals referred for echocardiography; however, no data from relevant control groups was reported^5^. Sangal and colleagues also reported that interferon-based treatment in 26 individuals was associated with higher PASP (6.00 mmHg, 95% CI 0.09–11.90) adjusted for age and sex, though the confidence intervals were wide. In contrast, we did not observe an association between interferon exposure and PASP, though our cohort also suffers from a small sample size receiving inteferon (n = 48).

The relationship between treatment of HIV with ART and PASP remains unclear. In 2004, Zuber et al. reported that receipt of NRTI-based regimens or highly-active ART was associated with a longitudinal PASP reduction of 3 mmHg and 21 mmHg, respectively^13^. Schwarze-Zander et al. and Reinsch et al. found no association between receipt of ART and echocardiographic PASP estimate^3,11^. Our findings appear to confirm the latter two studies, though further investigation of the effects of more contemporary ART regimens is warranted.

Although this analysis has many strengths, its limitations should also be discussed. The primary limitation is the lack of invasively confirmed PH. We used an echocardiographic definition of PH, which may over or underestimate true pulmonary pressures. This retrospective study uses a cohort of veterans who were referred for a clinically-indicated echocardiogram which may not be generalizable to the US population. The VA population is predominantly male, though our female sample compares favorably in size to other similar reports. Echocardiograms in this study were not interpreted in a core laboratory or performed according to a standard protocol. We excluded subjects in whom PASP could not be estimated due to an inadequate tricuspid regurgitant jet. We decided not to impute normal values for these individuals because the prevalence of invasively confirmed PH is relatively high among patients with an absent tricuspid regurgitant velocity^14^. Though this is a retrospective analysis, the interval between echocardiogram and CD4 + T-cell count and HIV viral load was short (median [Q1, Q3]: − 12 [− 46, 24] days), supporting the clinical utility of our findings. Given these limitations and others discussed below, combined with the cross-sectional nature of our study, these findings are considered hypothesis-generating rather than suggestive of causality.

The data collection period for our study spans clinical practice changes in HIV care. For example, a substantial proportion of the cohort was not receiving ART, likely reflecting the ART guidelines prior to the 2011 endorsement of universal treatment of HIV. While the cohort captures a period of time after the first integrase inhibitor, raltegravir, became available on the VA formulary, the data regarding integrase inhibitors is too limited (122 subjects received raltegravir at any point and dates were not available) as to preclude its inclusion in the regression. Given that nearly every current first-line ART regimen today includes an integrase inhibitor, the effect of more potent treatments on PASP requires further study in more contemporary cohorts, which is an area of ongoing study.

The analysis of chronic HCV infection in this study may be limited due to the complexity of retrospective classification. HCV testing was far more remote from baseline echocardiogram than HIV testing (median [Q1, Q3]: − 674 [− 1509, − 119] days). However, in spite of these limitations, when we defined chronic HCV infection with high specificity we observed a modest association between HIV/HCV coinfection and PASP. The effect of chronic HCV infection warrants further study, especially in the era of direct-acting antiretroviral agents^15^.

In addition, we did not account for presence of interstitial lung disease in our analyses. Diagnostic codes for interstitial lung disease have low sensitivity and specificity^16^. Thus, assigning patients with this diagnosis would require further refinement of our natural language processing tool, which has not yet been undertaken. Thus, residual confounding due to the impact of interstitial lung disease may remain in our results.

The modest association of HIV/HCV coinfection with PASP does not appear to be of clinical importance. The burden of echocardiographic pulmonary hypertension is high among HIV-infected veterans but does not appear to be strongly mediated by traditionally measured viral factors and the presence of HCV coinfection does not appreciably increase echocardiographic PH risk. Our data suggest that lower CD4 + T-cell counts places PLWH at higher risk for elevated pulmonary arterial pressure, which supports the notion that an immunological mechanism, rather than a direct viral effect, may influence PASP among veterans with HIV infection. We view these findings as hypothesis generating which warrant further investigation in prospective studies.

## Methods

### Design, cohort, inclusion/exclusion criteria

We performed a series of cross-sectional analyses using data from the VACS which enrolls HIV-infected veterans matched 1:2 on age, race, sex, geographic site to uninfected veterans from Veterans Affairs (VA) infectious disease and primary care clinics. HIV status was determined by medical record review by trained VACS recruiters at the time of enrollment^17^.

VACS participants enrolled between April 2003 through September 2012 with at least one echocardiographic PASP estimate were included in the analysis.

**Figure 1:**
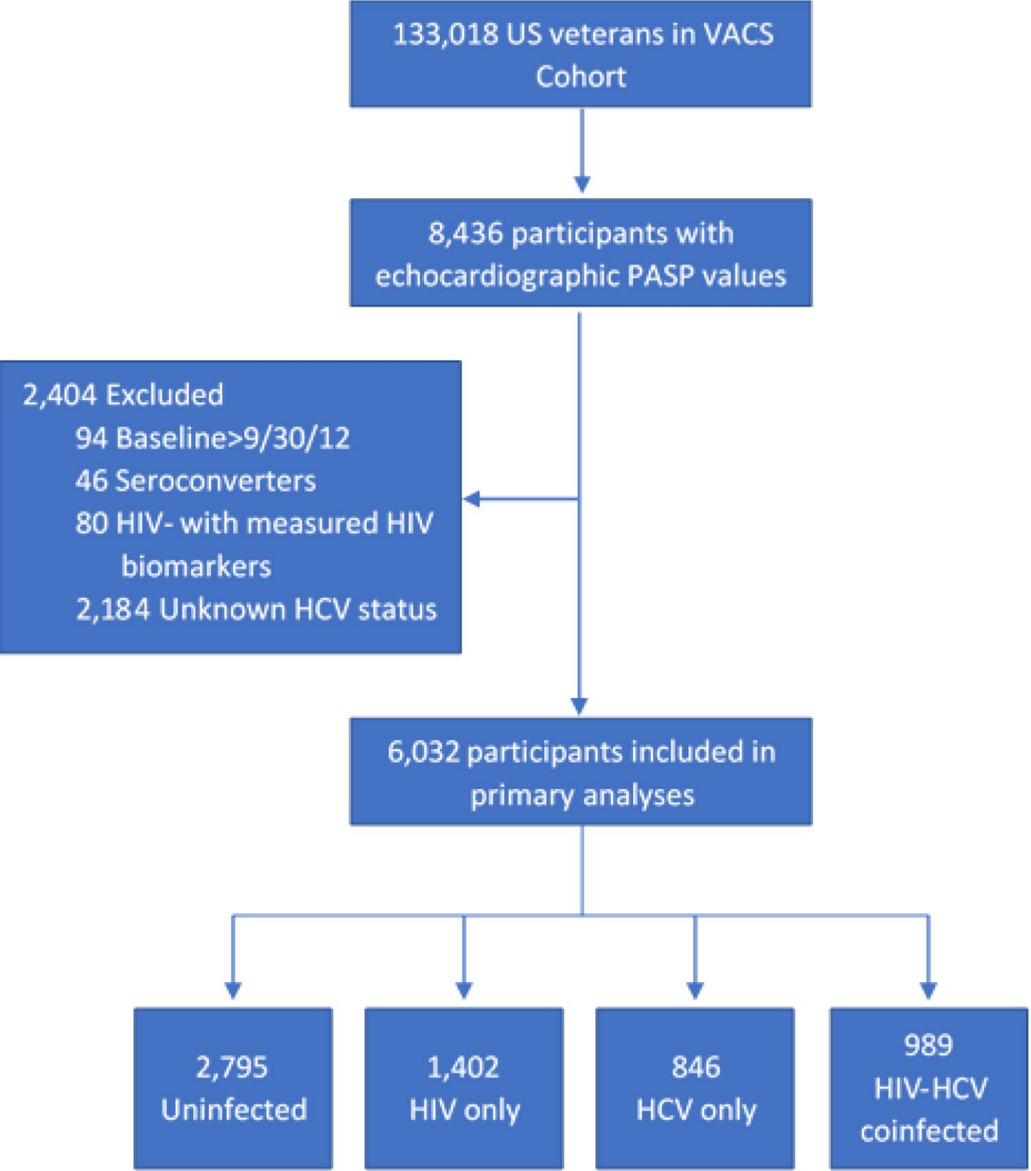
Flowchart of participants in primary analyses. *VACS* Veterans Aging Cohort Study, *PASP* pulomary artery systolic pressure, *HCV* hepatitis C virus.

From 8436 VACS participants with measured PASP, we excluded individuals whose echocardiogram was performed after September 30, 2012 and those who were classified as HIV uninfected but who had had laboratory results measuring HIV biomarkers. In primary analyses, we additionally excluded participants whose HCV status could not be determined for a final sample size of 6032 (Fig. 1). The institutional review boards (IRB) at Vanderbilt University Medical Center, Tennessee Valley Healthcare System, Yale University, and West Haven VA Medical Center approved this study. No informed consent was required; all methods were carried out in accordance with relevant guidelines and regulations.

### Echocardiographic data collection

Echocardiographic data were extracted for the first echocardiogram performed in the VA system on or after April 1, 2003^2^. Estimated PASP was extracted from echocardiogram reports using a previously validated custom rule based information system in the VA electronic health record^18^. The VA echocardiographic laboratories follow American Society of Echocardiography guidelines for estimating PASP using the simplified Bernoulli equation^19^. The interpreting physician’s estimation of right atrial pressure is incorporated into the extracted PASP value. PASP estimates considered to be outside a physiologic range (< 12 mmHg or > 152 mmHg) were excluded.

### Classification of HIV and HCV infection

HIV status was determined based on at least one inpatient or two or more outpatient International Classification of Disease, 9^th^ revision (ICD-9) codes for HIV and if the participant was included in the VA Immunology Case Registry^20^. HCV status was considered positive if any of the following criteria were met: positive qualitative HCV test, detectable HCV viral load, or positive antibody test, or receipt of telaprevir, ribavirin, or interferon therapy. In primary analyses, individuals were considered to be HCV negative only in the presence of a negative qualitative test.

### ART and HCV medication classification

HIV treatment regimens were categorized by class components as follows: nonnucleoside reverse transcriptase inhibitors plus nucleoside reverse transcriptase inhibitors alone (NNRTI + NRTI), protease inhibitors plus nucleoside reverse transcriptase inhibitors alone (PI + NRTI), other antiretroviral therapy (ART) regimens , and no ART. HCV medications included in the analysis were standard interferon alpha and pegylated interferon alpha (analyzed together as one variable due to low total numbers), ribavirin, and the single direct-acting antiviral agent used during the study period, telepravir.

### Collection of covariates

Age, sex, and race/ethnicity data were obtained from administrative records. Status of comorbidities including coronary heart disease, heart failure, chronic obstructive pulmonary disease, stroke, and substance abuse including smoking status, alcohol consumption and cocaine use, was determined using ICD-9 codes, laboratory or physiologic measurements, or self-reported data closest to the date of first echocardiogram, using definitions validated in previous VACS publications^4,18,21^. The status of comorbidities with available physiologic measurements or biomarkers, including anemia, diabetes mellitus, hypertension, chronic kidney disease, chronic liver disease, and dyslipidemia, were established using blood hemoglobin concentration, fasting insulin levels, outpatient blood pressure readings, estimated glomerular filtration rate (eGFR), fibrosis-4 score (FIB-4), and lipid panels, respectively^22^. Prevalent portal hypertension was defined as the presence of one inpatient or two outpatient ICD-9 codes of 572.3. Comorbidities were considered prevalent if the definitions were satisfied at any time prior to the echocardiogram or within 180 days after.

HIV-specific factors, including lifetime nadir CD4 + T-cell count, closest CD4 + T-cell count to echocardiogram, closest HIV viral load to echocardiogram, and ART status, were also collected. ART status required exposure within 180 days prior to the echocardiogram or within 7 days after. HCV-specific factors were collected including HCV antibody testing, HCV qualitative and quantitative polymerase chain reaction, and medications targeting HCV.

### Statistical analysis

Based on their status at the time of echocardiogram, participants were assigned to one of four categories: uninfected, HIV mono-infected, HCV mono-infected, and HIV/HCV coinfected. Descriptive statistics were generated in each group as mean, standard deviation, and median for continuous variables, and as N (%) for categorical variables. Differences in baseline characteristics across infection groups were tested using Kruskal–Wallis tests for continuous variables and chi-square tests for categorical variables.

We performed multiple linear regression to determine whether HIV/HCV mono-infection or coinfection was associated with higher PASP compared to neither infection. Models were adjusted for age, sex, race/ethnicity, body mass index (BMI), hypertension, diabetes mellitus, dyslipidemia, smoking status, alcohol abuse, cocaine abuse, eGFR, hemoglobin, and history of heart failure, heart disease, or stroke; covariates were selected a priori by identifying known or likely demographic and pathologic confounders. Next, we restricted models to HIV + subjects to assess the association of HCV coinfection, higher HIV viral load, lower CD4 + T-cell count, and ART with PASP levels. Similarly, models were limited to those with chronic HCV infection to determine whether HIV coinfection, higher HCV viral load, or interferon use was associated with increasing PASP. In models limited to HIV + subjects or HCV + participants, our sample size was reduced (N = 2391 and 1835, respectively), so models were adjusted for age, sex, race/ethnicity, and history of heart failure; adjustment for the full set of covariates would result in overfitting the model.

Finally, we performed multivariable-adjusted logistic regression analyses using echocardiographic PH as the dependent variable. Three sets of logistic regression analyses were performed defining echocardiographic PH as PASP > 35, > 40, or > 60 mmHg, respectively. These cutoffs reflect commonly used clinical thresholds for defining echocardiographic PH (35 and 40 mmHg) and a highly specific threshold (60 mmHg) which reduces misclassification given the relative imprecision of echocardiographic estimates of PASP. In these analyses, HIV/HCV status served as the exposure of interest and these models were adjusted for age, sex, race/ethnicity, BMI, hypertension, diabetes mellitus, dyslipidemia, smoking status, alcohol abuse, cocaine abuse, eGFR, hemoglobin, and history of heart failure, heart disease, or stroke.

Studying chronic HCV retrospectively has several challenges: 1) HCV screening is not performed in every subject, 2) confirmatory diagnostic testing is not performed in every subject with positive screening, 3) there is variation in confirmatory testing modalities, 4) testing may be temporally remote from the echocardiographic PASP assessment, and 5) HCV may be acquired after initial screening^17^. Due to the challenge of classifying chronic HCV infection retrospectively, primary analyses excluded those without a confirmed HCV status, sacrificing overall number of subjects in favor of improved diagnostic accuracy.

In sensitivity analyses, we returned VACS participants with measured PASP but without a confirmed HCV status to the “HCV − “ group corresponding to their confirmed HIV status (i.e. HIV-uninfected participants with an unknown HCV status were included in the “uninfected” group while HIV + participants with unknown HCV status were included in the “HIV mono-infected” group.) This choice biases toward the null of no association since individuals who are HCV positive would be misclassified as HCV negative. The methods for descriptive statistics and multiple linear regression analyses were repeated as described above in this larger sample of 8216 VACS participants. Given the null results of logistic regression analyses in the primary sample, logistic regression analyses were not repeated in sensitivity analyses.

In exploratory analyses, we performed linear and logistic regression to assess whether prevalent portal hypertension was associated with increased PASP and/or echocardiographic PH in individuals with HIV/HCV coinfection. Models were unadjusted due to the small number of individuals with HIV/HCV coinfection and prevalent portal hypertension.

Missing data were multiply imputed to create 5 complete datasets using predictive mean matching for continuous variables to produce biologically plausible values, while the discriminant function with a non-informative Jeffrey’s prior was used to impute categorical variables. All analyses were performed in SAS version 9.4 (Cary, NC) and a two-sided *p* value < 0.05 was considered statistically significant.

## Supplementary information

Supplementary Information

## Data Availability

The datasets generated during and/or analyzed during the current study are not publicly available due to the identifiability and sensitive nature of the datasets (electronic medical records) but are available in a deidentified fashion from the corresponding author on reasonable request following approval by the respective IRBs.
