# Supplementary material for: HIV- and HCV-specific markers and echocardiographic pulmonary artery systolic pressure among United States veterans
Source: Sci Rep. 2020 Oct 30;10:18729. doi: 10.1038/s41598-020-75290-4 (PMC7599329; doi:10.1038/s41598-020-75290-4)
Supplement: Supplementary file 1 — Supplementary Information [file 41598_2020_75290_MOESM1_ESM.docx]

Supplementary Information

**HIV- AND HCV-SPECIFIC MARKERS AND ECHOCARDIOGRAPHIC PULMONARY ARTERY SYSTOLIC PRESSURE AMONG US VETERANS**

Courtney E. Zola, MD^1†^ ([courtney.zola@gmail.com](mailto:courtney.zola@gmail.com)), Meredith S. Duncan, MA^2†^ ([meredith.s.duncan@vumc.org](mailto:meredith.s.duncan@vumc.org)), Kaku So-Armah, PhD^3^ ([kaku@bu.edu](mailto:kaku@bu.edu)), Kristina A. Crothers, MD^4^ ([KCrothers@medicine.washington.edu](mailto:KCrothers@medicine.washington.edu)), Adeel A. Butt, MD^5^ ([aab2005@qatar-med.cornell.edu](mailto:aab2005@qatar-med.cornell.edu)), Cynthia L. Gibert, MD^6^ ([celgibert@gmail.com](mailto:celgibert@gmail.com)), Joon Woo W. Kim, MD^7^ ([joon.kim3@va.gov](mailto:joon.kim3@va.gov)), Joseph K. Lim, MD^8^ ([joseph.lim@yale.edu](mailto:joseph.lim@yale.edu)), Vincent Lo Re III, MD^9^ ([vincentl@pennmedicine.upenn.edu](mailto:vincentl@pennmedicine.upenn.edu)), Hilary A. Tindle, MD^10,11^ ([hilary.tindle@vumc.org](mailto:hilary.tindle@vumc.org)), Matthew S. Freiberg, MD^2, 10, 11^ ([matthew.s.freiberg@vumc.org](mailto:matthew.s.freiberg@vumc.org)), Evan L. Brittain, MD^2,11^* ([evan.brittain@vumc.org](mailto:evan.brittain@vumc.org))

†These authors contributed equally and are co-first authors of this manuscript

*Corresponding author

^1^ Division of Infectious Disease, Department of Medicine, Vanderbilt University Medical Center, Nashville, Tennessee, USA

^2^ Division of Cardiovascular Medicine, Vanderbilt University Medical Center, Nashville, TN, USA

^3^ Boston University School of Medicine, Section of General Internal Medicine, Boston, MA, USA

^4^Department of Medicine, University of Washington School of Medicine, Seattle, WA, USA

^5^ VA Pittsburgh Healthcare System, Pittsburgh, PA, USA and Weill Cornell Medical College, New York, NY, USA and Doha, Qatar

^6^ Department of Medicine, George Washington University, Washington, DC, USA

^7^Department of Medicine, James J. Peters VA Medical Center, Icahn School of Medicine at Mt. Sinai, New York City, New York, USA

^8^ Department of Medicine, Yale University School of Medicine, New Haven, CT, USA

^9^ Division of Infectious Disease, Department of Medicine and Center for Clinical Epidemiology and Biostatistics, Perelman School of Medicine, University of Pennsylvania, Philadelphia, PA, USA

^10^Geriatric Research Education and Clinical Centers (GRECC), Veterans Affairs Tennessee Valley Healthcare System, Nashville, TN, USA

^11^Department of Medicine, Vanderbilt University Medical Center, Nashville, Tennessee, USA

Table of Contents

[Supplementary Table Legends 2](#_Toc35962656)

[Table S1. Sensitivity Analysis with Unknown HCV status classified as HCV- 3](#_Toc35962657)

[Table S2. Regression Analysis with Indeterminate HCV status set to HCV- 4](#_Toc35962658)

[Table S3. Association of HIV/HCV Viral Markers and Medication Regimens with PASP (mmHg); Indeterminate HCV status set to HCV- 5](#_Toc35962659)

#

# Supplementary Table Legends

**Table S1.** Sensitivity Analysis with Unknown HCV status classified as HCV-

Data presented as mean±standard deviation or n(%).

Abbreviations: BMI, body mass index; HF, heart failure; EF, ejection fraction; CHD, coronary heart disease; HTN*,* hypertension; COPD, chronic obstructive pulmonary disease; eGFR, estimated glomerular filtration rate; FIB-4, fibrosis 4 score; ART, anti-retroviral therapy; NRTI, nucleoside reverse transcriptase inhibitor; PI, protease inhibitor; NNRTI, non-nucleoside reverse transcriptase inhibitor

* All characteristics were significantly different across HIV/HCV groups, via Wilcoxon tests or χ^2^ test except PASP>40 (p=0.7325), receipt of PAH medications (p=0.4630), COPD (p=0.1806), history of stroke (p=0.1297), CD4 Nadir (p=0.3114), and HCV viral load (p=0.2710).

† All variables had complete data except BMI (available on 4539 uninfected, 1832 HIV mono-infected, 844 HCV mono-infected, 987 co-infected), hypertension (available on 4542 uninfected, 1835 HIV mono-infected, 845 HCV mono-infected, 989 co-infected), smoking status (available on 4149 uninfected, 1622 HIV mono-infected, 804 HCV mono-infected, 923 co-infected), dyslipidemia (available on 4357 uninfected, 1732 HIV mono-infected, 815 HCV mono-infected, 962 co-infected), eGFR (available on 4500 uninfected, 1824 HIV mono-infected, 846 HCV mono-infected, 989 co-infected), FIB-4 (available on 4325 uninfected, 1506 HIV mono-infected, 836 HCV mono-infected, 872 co-infected), hemoglobin (available on 4484 uninfected, 1825 HIV mono-infected, 846 HCV mono-infected, 987 co-infected), HIV RNA (available on 1498 HIV mono-infected, 873 co-infected), CD4 cell count (available on 1495 HIV mono-infected, 874 co-infected), CD4 nadir (available on 1495 HIV mono-infected, 874 co-infected), and HCV RNA (available on 655 HCV mono-infected, 795 co-infected).

**Table S2.** Regression Analysis with Indeterminate HCV status set to HCV-

Abbreviations: BMI, body mass index; HF, heart failure; EF, ejection fraction; CHD, coronary heart disease; HTN*,* hypertension; COPD, chronic obstructive pulmonary disease; eGFR, estimated glomerular filtration rate; FIB-4, fibrosis 4 score; ART, anti-retroviral therapy; NRTI, nucleoside reverse transcriptase inhibitor; PI, protease inhibitor; NNRTI, non-nucleoside reverse transcriptase inhibitor

* All estimates are from a multiple linear regression adjusted for all listed variable

**Table S3.** Association of HIV/HCV Viral Markers and Medication Regimens with PASP (mmHg); Indeterminate HCV status set to HCV-

Abbreviations: HCV, hepatitis C virus; PASP, pulmonary artery systolic pressure; CI, confidence interval; ART, anti-retroviral therapy; NRTI, nucleoside reverse transcriptase inhibitor; PI, protease inhibitor; NNRTI, non-nucleoside reverse transcriptase inhibitor

* Adjusted for: age, sex, race/ethnicity, and heart failure

# **Table S1.** Sensitivity Analysis with Unknown HCV status classified as HCV-

|  | **Uninfected**  **(n = 4,544)** | **HIV Only**  **(n = 1,837)** | **HCV Only**  **(n = 846)** | **HIV/HCV**  **(n = 989)** |
| --- | --- | --- | --- | --- |
| Age, years | 58.3 ± 9.9 | 57.5 ± 11.0 | 56.2 ± 6.1 | 56.2 ± 7.4 |
| Race C*aucasian*  *African American*  *Hispanic*  *Other* | 1836 (40.4)  2145 (47.2)  457 (10.1)  106 (2.3) | 783 (42.6)  860 (46.8)  131 (7.1)  63 (3.4) | 221 (26.1)  545 (64.4)  73 (8.6)  7 (0.8) | 283 (28.6)  598 (60.5)  92 (9.3)  16 (1.6) |
| Male Sex | 4394 (96.7) | 1789 (97.4) | 834 (98.6) | 966 (97.7) |
| BMI, kg/m^2^† | 30.2 ± 6.9 | 26.0 ± 5.7 | 27.8 ± 6.1 | 25.5 ± 5.3 |
| PASP (mmHg) | 36.0 ± 14.1 | 36.0 ±14.8 | 36.6 ± 14.6 | 37.0 ± 16.2 |
| PASP > 40 mmHg^*^ | 1224 (26.9) | 498 (27.1) | 237 (28.0) | 282 (28.5) |
| PAH Medication^*^ | 30 (0.7) | 19 (1.0) | 7 (0.8) | 9 (0.9) |
| Heart Failure Status *None*  *HF Preserved EF*  *HF, EF between 40 & 50*  *HF Reduced EF*  *HF, No EF* | 2956 (65.1)  234 (5.2)  91 (2.0)  301 (6.6)  962 (21.2) | 1287 (70.1)  71 (3.9)  42 (2.3)  145 (7.9)  292 (15.9) | 597 (70.6)  45 (5.3)  12 (1.4)  58 (6.9)  134 (15.8) | 707 (71.5)  60 (6.1)  27 (2.7)  75 (7.6)  120 (12.1) |
| CHD History | 2407 (53.0) | 768 (41.8) | 387 (45.7) | 318 (32.2) |
| Hypertension†  *Absent*  *Controlled HTN*  *Uncontrolled HTN* | 168 (3.7)  2922 (64.3)  1452 (32.0) | 188 (10.3)  1188 (64.7)  459 (25.0) | 30 (3.6)  506 (59.9)  309 (36.6) | 75 (7.6)  616 (62.3)  298 (30.1) |
| COPD^*^ | 1560 (34.3) | 601 (32.7) | 312 (36.9) | 348 (35.2) |
| Stroke History^*^ | 344 (7.6) | 118 (6.4) | 69 (8.2) | 60 (6.1) |
| Diabetes | 2063 (45.4) | 638 (34.7) | 390 (46.1) | 385 (38.9) |
| Smoking status† *Never*  *Former*  *Current* | 1420 (34.2)  1109 (26.7)  1620 (39.1) | 530 (32.7)  362 (22.3)  730 (45.0) | 143 (17.8)  182 (22.6)  479 (59.6) | 200 (21.7)  226 (24.5)  497 (53.9) |
| Dyslipidemia† | 2622 (60.2) | 1202 (69.4) | 472 (57.9) | 635 (66.0) |
| Alcohol Abuse | 1422 (31.3) | 555 (30.2) | 545 (64.4) | 535 (54.1) |
| Cocaine Abuse | 697 (15.3) | 390 (21.2) | 384 (45.4) | 443 (44.8) |
| eGFR (mL/min/1.73 m²)† | 79.3 ± 31.0 | 79.8 ± 35.1 | 84.9 ± 46.9 | 82.3 ± 39.1 |
| FIB-4† | 1.5 ± 1.4 | 1.8 ± 2.6 | 2.7 ± 3.5 | 3.2 ± 3.7 |
| Hemoglobin (mg/dL) † | 13.6 ± 1.9 | 13.0 ± 2.1 | 13.3 ± 2.1 | 12.9 ± 2.1 |
| HIV RNA (copies/mL) †  *Median* |  | 40,886.0 ± 252,094.1  75.0 |  | 24,558.8 ± 85,996.9  75.0 |
| CD4+ T-Cell Count†  *Median* |  | 444.4 ± 301.2  389.0 |  | 417.2 ± 275.8  364.0 |
| Nadir CD4+ T-Cell Count^*,^†  *Median* |  | 260.2 ± 211.6  217.0 |  | 241.9 ± 184.1  209.0 |
| ART Regimen *NRTI+PI*  *NRTI+NNRTI*  *Other regimen*  *No ART* |  | 362 (19.7)  262 (14.3)  826 (45.0)  387 (21.1) |  | 207 (20.9)  106 (10.7) 504 (51.0)  172 (17.4) |
| HCV RNA (copies/mL)^*,^ †  *Median* |  |  | 1,769,144.4 ± 3,806,313.0  500,000 | 2,550,873.6 ± 5,854,693.8  604,000 |
| HCV Treatment *Interferon*  *Ribavirin*  *Telaprevir* |  |  | 24 (2.8)  21 (2.5)  1 (0.1) | 21 (2.1)  21 (2.1)  0 (0.0) |

# **Table S2.** Regression Analysis with Indeterminate HCV status set to HCV-

| **Variables** | **β-estimate (mmHg)  [95% CI]^*^** | **P-value** |
| --- | --- | --- |
| HIV/HCV Status  Uninfected  HIV mono-infected  HCV mono-infected  Co-infected | Ref  0.004 [-0.93, 0.94]  0.45 [-0.66, 1.57]  1.12 [0.02, 2.21] | ---  0.9940  0.4267  0.0459 |
| Age, 10 years | 0.22 [-0.21, 0.65] | 0.3105 |
| Race  White  African American  Hispanic  Other | Ref  2.01 [1.19, 2.84]  -3.33 [-4.58, -2.07]  0.13 [-2.20, 2.46] | ---  <.0001  <.0001  0.9107 |
| Male Sex | 7.42 [5.32, 9.52] | <.0001 |
| BMI | 0.04 [-0.02, 0.10] | 0.2145 |
| Heart Failure Status  HF Preserved EF  HF with EF between 40 & 50  HF Reduced EF  No EF  No HF | 7.82 [6.17, 9.47]  6.44 [3.89, 8.90]  5.76 [4.36, 7.17]  7.93 [6.89, 8.97]  Ref | <.0001  <.0001  <.0001  <.0001  --- |
| History of CHD | 1.03 [0.25, 1.80] | 0.0096 |
| Hypertension  No Hypertension  Controlled HTN  Uncontrolled HTN | Ref  0.31 [-1.26, 1.89]  1.65 [-0.02, 3.31] | ---  0.6962  0.0527 |
| COPD | 1.96 [1.19, 2.72] | <.0001 |
| Stroke History | -1.20 [-2.55, 0.16] | 0.0839 |
| Diabetes Mellitus | 0.91 [0.14, 1.67] | 0.0198 |
| Dyslipidemia | -0.47 [-1.23, 0.28] | 0.2203 |
| Smoking Status  Current  Former  Never | 0.52 [-0.43, 1.46]  0.71 [-0.36, 1.78]  Ref | 0.2835  0.1906  --- |
| Alcohol Use | 0.19 [-0.69, 1.07] | 0.6690 |
| Cocaine Use | -1.55 [-2.57, -0.52] | 0.0031 |
| eGFR (mL/min/1.73 m²) | -0.03 [-0.04, -0.01] | <.0001 |
| FIB-4 | 0.31 [0.16, 0.46] | <.0001 |
| Hemoglobin (mg/dL) | -0.76 [-0.95, -0.57] | <.0001 |

# **Table S3.** Association of HIV/HCV Viral Markers and Medication Regimens with PASP (mmHg); Indeterminate HCV status set to HCV-

| **Variables Among All HIV+** | **β-estimate (mmHg)  [95% CI]^*^** | **P-value** |
| --- | --- | --- |
| HCV positive status | 1.46 [0.26, 2.66] | 0.0174 |
| HIV viral load (10,000 copies/mL) | -0.02 [-0.05, 0.02] | 0.3547 |
| CD4+ T-cell count (200 cells/mm^3^) | -0.73 [-1.16, -0.29] | 0.0011 |
| ART Regimen |  |  |
| NRTI+PI vs. No ART | 0.66 [-1.28, 2.59] | 0.5060 |
| NRTI+NNRTI vs. No ART | 0.55 [-1.57, 2.67] | 0.6125 |
| Other ART vs. No ART | -0.90 [-2.59, 0.79] | 0.2970 |
| **Variables Among All HCV Cases** | **β-estimate (mmHg)**  **[95% CI]^*^** | **P-value** |
| HIV positive status | 0.77 [-0.60, 2.13] | 0.2719 |
| HCV viral load (10,000 copies/mL) | 0.001 [-0.001, 0.003] | 0.2513 |
| Interferon exposure | 0.44 [-3.95, 4.84] | 0.8429 |
